# Case report: Autosomal recessive bestrophinopathy with macular cysts and MNV over 13-year follow-up

**DOI:** 10.3389/fgene.2022.1045145

**Published:** 2022-11-15

**Authors:** Lei Zhang, Hai-Yan Wang, Wei Jia, Ru Wang, Yu-Sheng Wang, Yang-Yang Cui

**Affiliations:** ^1^ Shaanxi Eye Hospital, Xi’an People’s Hospital (Xi’an Fourth Hospital), Xi’an, China; ^2^ Department of Ophthalmology, Xijing Hospital, Xi’an, China

**Keywords:** recessive bestrophinopathy, *BEST1* gene, macular dystrophy, macular neovascularization, macular cysts

## Abstract

**Purpose:** To describe the phenotype and genotype of a patient with autosomal recessive bestrophinopathy (ARB) over a 13-year follow-up period.

**Methods:** The phenotype of the subject was described after a complete ophthalmological examination, which included fundus photography, optical coherence tomography (OCT), fundus autofluorescence, fluorescein angiography (FA), indocyanine green angiography (ICGA), electroretinogram (EOG), electroretinography (ERG), and multifocal electroretinogram (mfERG). Genetic analyses were carried out by screening the variations *via* whole-exome sequencing.

**Results:** This patient presented with retinoschisis and cystic changes when he was 7 years old and was diagnosed with X-linked retinoschisis. In the 13th year after the first presentation, enlarged macular cysts with retinoschisis, macular neovascularization (MNV), and subretinal fluid were displayed on OCT. Autofluorescence showed hyperfluorescence corresponding to the area of retinal pigment epithelium (RPE) change. EOG showed no light peak, and the Arden ratio was less than 2.0. Whole-exome sequencing revealed compound heterozygous sequence variations (p. [Arg47Leu; Trp287*]) in the coding sequence of the BEST1 allele inherited from his parents. Thus, a diagnosis of ARB combined with secondary MNV was made.

**Conclusion:** Patients with compound heterozygous BEST1 mutations developed ARB, which could show significant retinoschisis at a young age. Genetic analyses, autofluorescence, and EOG are essential to diagnose ARB correctly in consequence of considerable phenotypic variations.

## Introduction

The bestrophin-1 (*BEST1*) gene (formerly known as *VMD2*) is located on the long arm of chromosome 11 (11q12) and encodes the bestrophin-1 protein located mainly in the retinal pigment epithelium (RPE). Its mutation may lead to a variety of ocular phenotypes known as “bestrophinopathies” (Khojasteh et al., 2021). Autosomal recessive bestrophinopathy (ARB) is caused by mutations in both alleles of the *BEST1* gene with either homozygous or compound heterozygous mutations. Burgess et al. (2008) first designated the constellation of findings in 2008 now known as ARB. High hyperopia, shallow anterior segment, angle-closure glaucoma, abnormal electroretinogram (EOG), intraretinal cystic changes, and concurrent subretinal fluid have been reported in ARB patients (Kalevar et al., 2018). Here, we report a patient who presented with retinoschisis and cystic changes in childhood and was identified as an individual with *BEST1* gene mutation 13 years later, and the clinical findings were consistent with ARB.

## Case report

A 20-year-old Chinese man presented with 2 weeks of blurred central vision in both eyes. His family history and social history were unremarkable. He was previously diagnosed with macular retinoschisis and choroidal neovascularization (CNV) in the left eye at the age of 7. The best-corrected visual acuity was presently 20/800 and 20/200 in his right and left eyes, respectively. Color vision testing showed a subclinical deficit. Bilateral ocular motility, intraocular pressure, night vision, and anterior segment examinations were within normal limits in both eyes.

**FIGURE 1:**
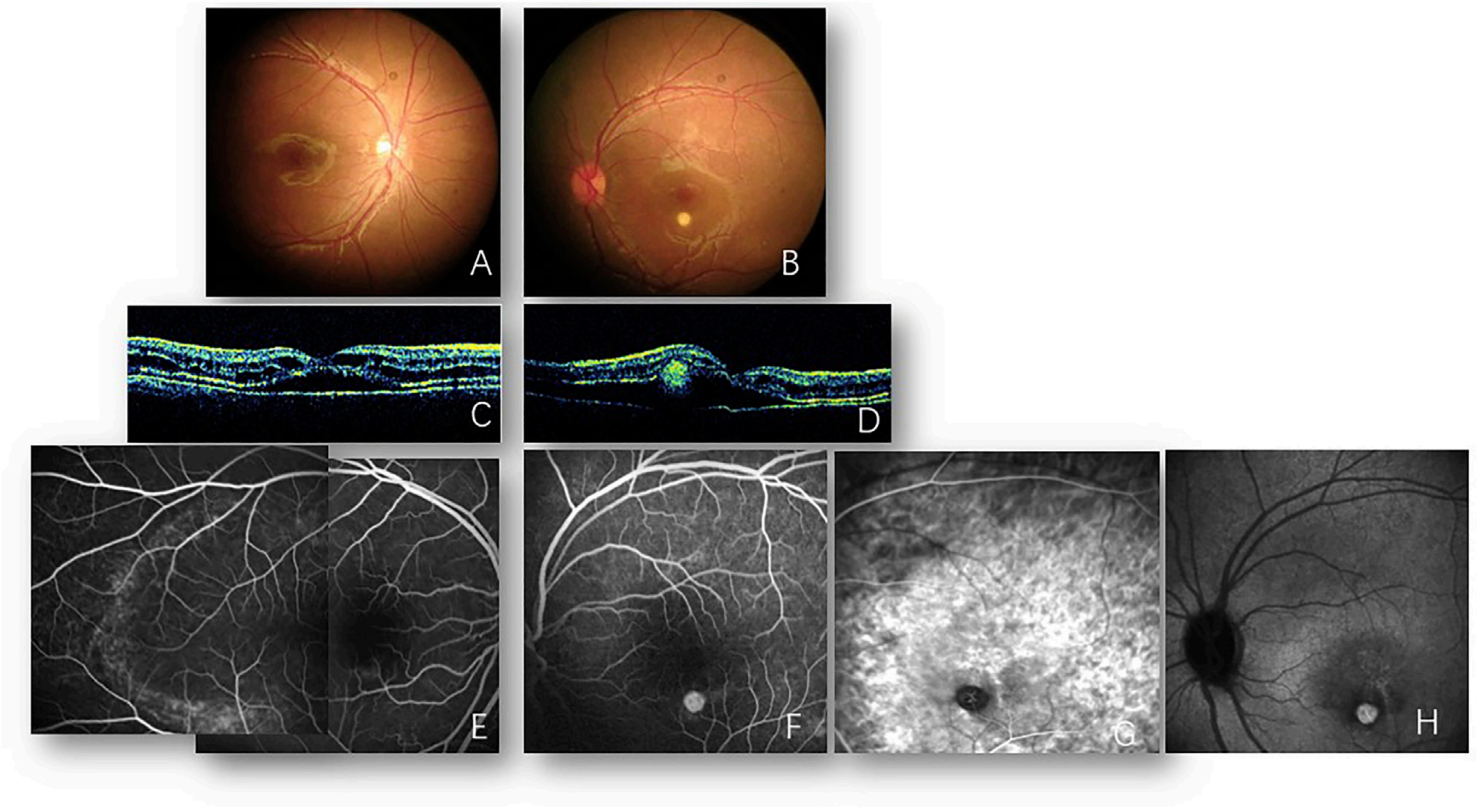
Images from 2008. Fundus photos of the right **(A)** and left **(B)** eyes reveal bilateral cystoid changes in the macula. A round yellowish subretinal lesion inferior to the macula of the left eye is also shown **(B)**. Optical coherence tomography of the right **(C)** and left **(D)** eyes reveals the splitting of retinal layers and foveal neurosensory retinal detachment with cystic changes. Subretinal hyperreflectivity lesion is also noted in the left eye **(B)**. Fluorescein angiography of the right eye **(E)** reveals multiple hyperfluorescent dots on the temporal macula. A round hyperfluorescent lesion is shown inferior to the macula of the left eye **(F)**, corresponding to the subretinal lesion visualized in **(B)**. Indocyanine green angiography of the left eye verified new vessels in the early **(G)** and late **(H)** phases.

Back in 2008, spoke-like appearances in the bilateral macula with a round yellowish lesion inferior to the left macula were shown in this patient’s medical records when 7 years old. Meanwhile, cystic changes and retinoschisis with subretinal fluid on optical coherence tomography (OCT) and slight macular fluorescein pooling in the late stage on fluorescein angiography (FA) were revealed bilaterally. Round choroidal neovascularization (CNV) was confirmed *via* OCT, FA, and indocyanine green angiography (ICGA) (Figure 1). Full-field electroretinogram (ERG) was nearly normal, but the P_1_ response density of the multifocal electroretinogram (mfERG) in ring 1 (macular area) was abnormal (Table 1). Unfortunately, due to the poor compliance of this patient at 7 years of age, EOG could not be obtained. At that time, he was diagnosed with X-linked retinoschisis (XLRS) and CNV in the left eye. Two years later, macular retinoschisis with cysts could still be visualized, and then the follow-up was lost until 2021.

**TABLE 1:** Changes in full-field electroretinogram (ERG), multifocal electroretinogram (mfERG), and electrooculogram (EOG) in this case that was followed up for 13 years.

Family number	Age (y)	Amplitudes of scotopic 0.01 ERG b-wave (μV) (OD,OS)	Amplitudes of scotopic 3.0 ERG a-wave (μV) (OD,OS)	Amplitudes of scotopic 3.0 ERG b-wave (μV) (OD,OS)	Amplitudes of photopic 3.0 ERG b-wave (μV) (OD,OS)	P1 response density of mfERG in ring 1 (macular area) (nv/degree^2^) (OD,OS)	Amplitudes of dark trough of EOG (μV) (OD,OS)	Amplitudes of light peak of EOG (μV) (OD,OS)	Arden ratio of EOG (OD,OS)
I	7	38.7, 61.4	85.21, 91.55	245.6, 321	39.6, 62.1	43, 62	——	——	——
I	20	66.2, 72	62.5, 88.5	163.9, 146.1	43.9, 62.1	12.6, 8.4	176.91, 168.2	247.07, 226.56	1.397, 1.374
I-1	48	149.5, 118.6	133.1, 206.8	358.1, 357.9	96.2, 96.1	57.4, 56.1	205.93, 258.81	380.86, 477.54	1.849, 1.845
I-2	45	88.7, 85.6	113.1, 121.1	248.9, 245.7	84.5, 87.9	31.2, 35.7	190.43, 147.31	434.57, 367.19	2.282, 2.493

Note: I, proband; I-1, father of proband; I-2, mother of proband; EOG, electroretinogram; ERG, full-field electroretinogram.

**FIGURE 2:**
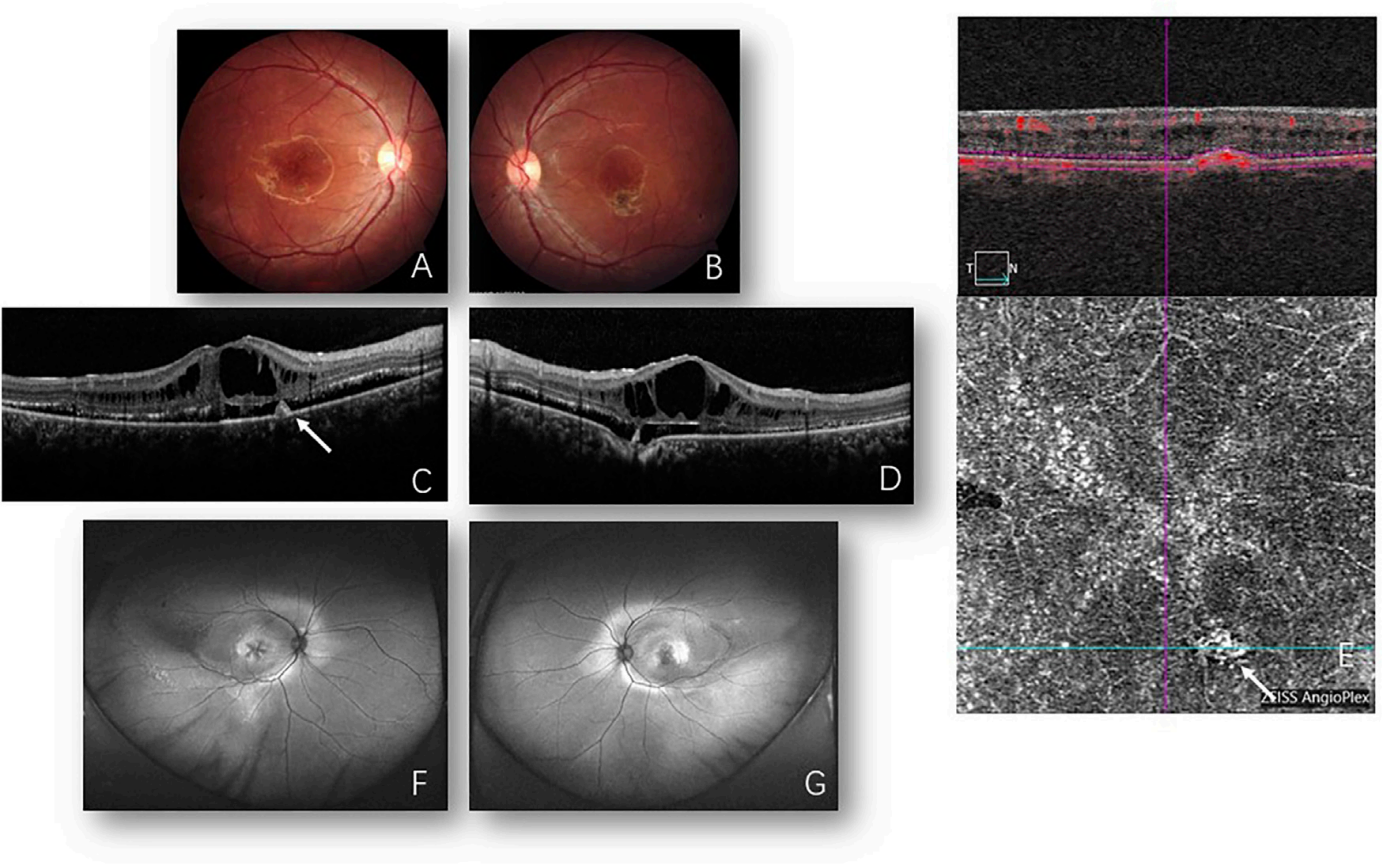
Images from 2021. Fundus photos of the right **(A)** and left **(B)** eyes reveal bilateral cystoid changes in the macula and bilateral pigmented lesion inferior to the macula. Optical coherence tomography (OCT) of the right **(C)** and left **(D)** eyes shows foveal retinoschisis with enlarged cysts presently when compared to back then in 2008 (Figures 1C, D) and “shaggy” photoreceptors in the subretinal space. Focal choroidal excavation is detected in the left eye **(D)**. Hyperreflectivity lesion in the RPE layer **(C)** corresponding to inactive macular neovascularization (MNV) is detected and confirmed by OCTA **(E)** (white arrow). Fundus autofluorescence **(F,G)** shows bilateral cystic changes in the macula and symmetric annular area of hyperfluorescence, suggesting retinal pigment epithelium (RPE) aberration in the posterior pole which extends nasal to the disc in both eyes mingled with subretinal deposits are present. Laser spots are distributed in the temporal peripheral retina in the right eye.

In 2021, 13 years after the first presentation, bilateral cystic changes in the macula and symmetric annular area of the retinal pigment epithelium (RPE) atrophy in the posterior pole were presented which extended nasal to the disc in both eyes mingled with subretinal deposits. Additionally, a pigmentary lesion inferior to the fovea was displayed in both eyes. Autofluorescence (Optos, Marlborough, MA, United States) showed hyperfluorescence corresponding to the area of RPE change. Enlarged macular cysts with retinoschisis and subretinal fluid (SRF) were displayed on OCT and focal choroidal excavation (FCE). Inactive macular neovascularization (MNV) was visualized inferior to the right macula on optical coherence tomography angiography (OCTA) (Figure 2). ERG studies showed decreased cone and rod responses, but no negative ERG could be detected. In addition, the P1 response density of mfERG in ring 1 was decreased, which was worse than that in 2008. The EOG showed an absence of light peak and the Arden ratio was less than 2.0 (Table 1). The fundus and EOG of the proband’s parents and little sister (aged 7 years) were all normal. Whole exome sequencing revealed the compound heterozygous sequence variations (p. [Arg47Leu; Trp287*]) in the coding sequence of the *BEST1* allele. Thus, a diagnosis of ARB combined with secondary MNV was made.

## Discussion

Pathogenic variants of *BEST1* result in functional disorders of the RPE, leading to the accumulation of subretinal fluid, bisretinoid N-retinyl-N-retinylidene ethanolamine (A_2_E), and lipofuscin, subsequently cause further RPE atrophy and photoreceptor cell loss. As a result, RPE irregularity, macular edema, retinoschisis, serous retinal detachment, and vitelliform deposits can be appreciated (Khojasteh et al., 2021). In this study, this ARB patient was characterized by subretinal fluid, hyperautofluorescence, abnormal EOG, macular retinoschisis, and young age at onset.

At the first presentation in 2008, this case was misdiagnosed as XLRS, most likely due to the rarity of this disease and the previous lack of full knowledge of the clinical features of this disease. At that time, there were also no fundus autofluorescence and EOG results, and the onset age was young. The typical clinical findings in patients with ARB often manifest as multifocal subretinal vitelliform deposits, diffuse SRF, and remarkable abnormalities on autofluorescence imaging (Kalevar et al., 2018), while it has not been reported that retinoschisis was present in any ARB patient before 2012 (Preising et al., 2012). In recent literature, ARB cases showed retinoschisis, macular cystic changes, and MNV (Khojasteh et al., 2021), which might be a possible pathogenic role of choroid in different stages of the disease (Grenga et al., 2016). In fact, misdiagnosis of ARB as XLRS has been reported in previous literature (Nakanishi et al., 2016; Habibi et al., 2019; Khojasteh et al., 2021). Definite diagnosis is usually based on genetic analysis, whereas clinical features such as early onset bilateral vitelliform depositions, cystic changes in retinal layers, abnormal hyperfluorescence as well as aberrant EOG could give some hint for ophthalmologists to make a differential diagnosis (Khojasteh et al., 2021). SRF and MNV are very rare in XLRS for differential diagnosis. Among auxiliary tests, visual electrophysiology plays an important role to differentiate XLRS and ARB, and b-wave amplitudes are reduced in XLRS, which could reveal a typical electronegative wave (Bowles et al., 2011). In comparison, negative b-wave would not be present in ARB patients, yet EOG is characterized by significant reduction or the absence of light rise and decreased Arden ratio. In our case, no negative b-waveform had ever showed up during the 13 years, but the EOG showed an absence of light rise and low Arden ratio when the proband was 21 years old. Other common macular retinoschisis diseases in children, such as Goldmann Favre syndrome, are caused by *NR2E3* gene mutation (Tsang and Sharma, 2018). Retinoschisis may also be secondary to pathological myopia, glaucoma, optic disc fovea, and other entities (Carr, 2020), but these diseases are rare in children and easy to distinguish from this case.

The age at onset in this case was young (7 years old). It had been reported that the youngest age at onset of ARB was in a 4-year-old boy, who presented with retinoschisis in the inner nuclear layer (INL) of both eyes, subretinal fluid, and MNV in the left eye (Khojasteh et al., 2021). However, he was followed up for only 3 years (Khojasteh et al., 2021). In this study, over a 13-year period, in addition to reduced best corrected visual acuity (BCVA), macular cystic, retinoschisis cavity, and subretinal fluid had progressed. Meanwhile, transmitted hyperfluorescence in the posterior pole appeared enhanced when compared with the late stage in 2008 on FA. The extensive dysfunction of photoreceptors and RPE in ARB corresponded with generalized lower responses in ERG than it did in 2008. Retinopathy involving the macula led to a further decrease in the amplitude of mfERG. Nevertheless, based on previous limited reports, whether vitelliform lesions in ARB cases advanced or regressed is still a controversial topic (Nakanishi et al., 2016; Luo et al., 2019). As far as we know, our patient has been followed up for the longest period when compared to previous reports.

Bestrophin-1 (BEST1) functions as calcium-activated anion channels and localizes to the basolateral aspect of the RPE (Nachtigal et al., 2020). Serous retinal detachments and retinoschisis suggest that the gene mutations affected chloride transport and calcium signal transduction, both of which were thought to be the basis of RPE ion transport and liquid homeostasis (Silva et al., 2013). These cystic changes could be attributed to structural abnormalities secondary to ionic transportation dysfunction of Müller cells and/or disrupted blood ocular barrier secondary to RPE cell ionic transportation dysfunction (Khojasteh et al., 2021). Müller cell dysfunction might have a significant role in retinoschisis and macular cysts combined with other possible causes, such as RPE dysfunction (Khojasteh et al., 2021). However, whether fluid existed in this cystic change needs to be confirmed by further basic research.

The compound heterozygous mutations p. Trp287*/p. Arg47Leu were detected in this proband. So far, more than 300 pathogenic mutations have been reported in the *BEST1* gene (Nachtigal et al., 2020). A cohort of Chinese ARB patients has shown that more than one-third of ARB mutations are located in exons 7 to 11, which encode the C-terminal half of the protein (Tian et al., 2017). Other mutations have been mainly clustered in the first transmembrane domain and the intracellular regions (Tian et al., 2017). These two mutations in our case were both in the hot regions of ARB (Tian et al., 2017). In this patient, p. Trp287* mutation was a nonsense mutation which was predicted to truncate the protein resulting in a loss of the cytoplasmic loop and has been detected and reported in two ARB patients (one Caucasian and one Asian) in the literature previously (MacDonald et al., 2012; Tian et al., 2017). In the two previously reported cases, the OCT image showed intraretinal cystoid space in the macula, but there were no SRF and MNV (MacDonald et al., 2012; Tian et al., 2017). The other mutation p. Arg47Leu mutation was not found in public databases, such as the Exome Variant Server and Genomes Database. However, it was predicted to be disease causing or probably damaging by three silico analysis programs (Polyphen2, Mutation Taster, and SIFT). Different variations in the same codon 47 (p. Arg47Cys) have been reported (Kinnick et al., 2011; Sodi et al., 2011). This novel mutation was located in a weakly conserved region of the protein, even though it was associated with a substitution involving two amino acids with different chemical properties (Kinnick et al., 2011; Sodi et al., 2011). This mutation as a tolerated change possibly affects protein function, with a potentially low pathogenic effect (Sodi et al., 2011). Thus, we believe p. Arg47Leu is still a pathogenic mutation. Many studies have attempted to determine the genotype–phenotype correlations of bestrophinopathy, but the evidence is limited. Even though Habibi et al. (2019) found that the homozygous *BEST1* mutation spectrum has certain clinical characteristics, the most common of these were extrafoveal and extramacular yellowish subretinal deposits. The homozygous variants of p.R255W had mainly macular edema and less subretinal fluid (Nakanishi et al., 2016). The study of a larger cohort is required to determine a definitive genotype–phenotype correlation. Cystic macular change in ARB may be difficult to treat, but occasionally oral acetazolamide has been useful. After 8 months of acetazolamide treatment in this case, the cystoid macular cavity in both eyes decreased (data not shown).

In conclusion, we report an ARB patient who presented visual symptoms at a young age and was followed up for 13 years. After the genetic test, the compound heterozygous mutation in the *BEST1* allele was found. Even though one locus was found to be novel, we think that the diagnosis of ARB in this case could be made in combination with the corresponding manifestations on clinical typical imaging examination. At 13 years after the first presentation, the manifestation of this case progressed and visual function decreased. Early accurate detection is essential to predict and monitor complications associated with this variant.

## Data Availability

The original contributions presented in the study are included in the article/supplementary material; further inquiries can be directed to the corresponding author.
